# Resistance of MMTV-NeuT/ATTAC mice to anti-PD-1 immune checkpoint therapy is associated with macrophage infiltration and Wnt pathway expression

**DOI:** 10.18632/oncotarget.28330

**Published:** 2022-12-20

**Authors:** Hongyan Yuan, Lu Jin, Handan Xiang, Anannya Bhattacharya, Philip E. Brandish, Gretchen Baltus, Alexander Tong, Changyan Zhou, Robert I. Glazer

**Affiliations:** ^1^Department of Oncology and Lombardi Comprehensive Cancer Center, Georgetown University Medical Center, Washington, DC 20007, USA; ^2^Discovery Immunology, Merck Research Institute, Boston, MA 02115, USA; ^3^Discovery Oncology, Merck Research Institute, Boston, MA 02115, USA; ^4^Bicycle Therapeutics, Lexington, MA 02421, USA

**Keywords:** PD-1, NeuT, Wnt, macrophages, mammary tumorigenesis

## Abstract

One of the central challenges for cancer therapy is the identification of factors in the tumor microenvironment that increase tumor progression and immune tolerance. In breast cancer, fibrosis is a histopathologic criterion for invasive cancer and poor survival that results from inflammatory factors and remodeling of the extracellular matrix to produce an immune tolerant microenvironment. To determine whether tolerance is associated with the immune checkpoint, Programmed Cell Death 1 (PD-1), NeuT/ATTAC mice, a conditional model of mammary fibrosis that we recently developed, were administered a murine-specific anti-PD-1 mAb related to pembrolizumab, and drug response was monitored by tumor development, imaging mass cytometry, immunohistochemistry and tumor gene expression by RNAseq. Tumor progression in NeuT/ATTAC mice was unaffected by weekly injection of anti-PD-1 over four months. Insensitivity to anti-PD-1 was associated with several processes, including increased tumor-associated macrophages (TAM), epithelial to mesenchymal transition (EMT), fibroblast proliferation, an enhanced extracellular matrix and the Wnt signaling pathway, including increased expression of Fzd5, Wnt5a, Vimentin, Mmp3, Col2a1 and Tgfβ1. These results suggest potential therapeutic avenues that may enhance PD-1 immune checkpoint sensitivity, including the use of tumor microenvironment targeted agents and Wnt pathway inhibitors.

## INTRODUCTION

It has become increasingly apparent that the cell-centric hallmarks of cancer originally proposed [1] are exceedingly more complex, and must now take into account the multi-faceted role of multiple cell types in the tumor microenvironment (TME) [2–5]. Although the TME has emerged as an important determinant of tumorigenesis as well as a plausible therapeutic target [6], understanding the specific cellular and molecular changes in the TME associated with breast cancer risk remains one of the overarching challenges for the prevention and treatment of this disease. Among the many stromal elements in the breast, adipose and fibrotic tissue comprise the largest components. Since stromal fibrosis is a histopathologic criterion of invasive breast cancer [7, 8], metastasis [9] and the development of precancerous lesions [10], identification of the signaling processes between tumor and stromal cells would greatly enhance our understanding of their roles in tumor progression. The TME undergoes extensive changes during the transition from pre-invasive to invasive ductal breast carcinoma as a result of the paracrine effects of inflammatory factors elicited by tumor cells and cancer-associated fibroblasts and macrophages [11, 12]. Stromal fibroblasts secrete chemokines such as Cxcl1 which enhances tumor progression [13–15] by inhibiting the adaptive immune response through recruitment and activation of regulatory T cells (Treg) and myeloid-derived suppressor cells (MDSC) [16–20].

There have been relatively few animal models to study the relationship between mammary fibrosis and tumorigenesis. To address this objective, we utilized the ErbB2 transgenic model, MMTV-NeuT/ATTAC, where stromal fibrosis can be induced conditionally by the targeted ablation of mammary adipose tissue [21]. Utilizing this more stringent tumor model to test its susceptibility to anti-PD-1 immunotherapy, we report the signaling processes associated with its lack of responsiveness.

## RESULTS

### Fibrosis and tumor development

**Figure 1:**
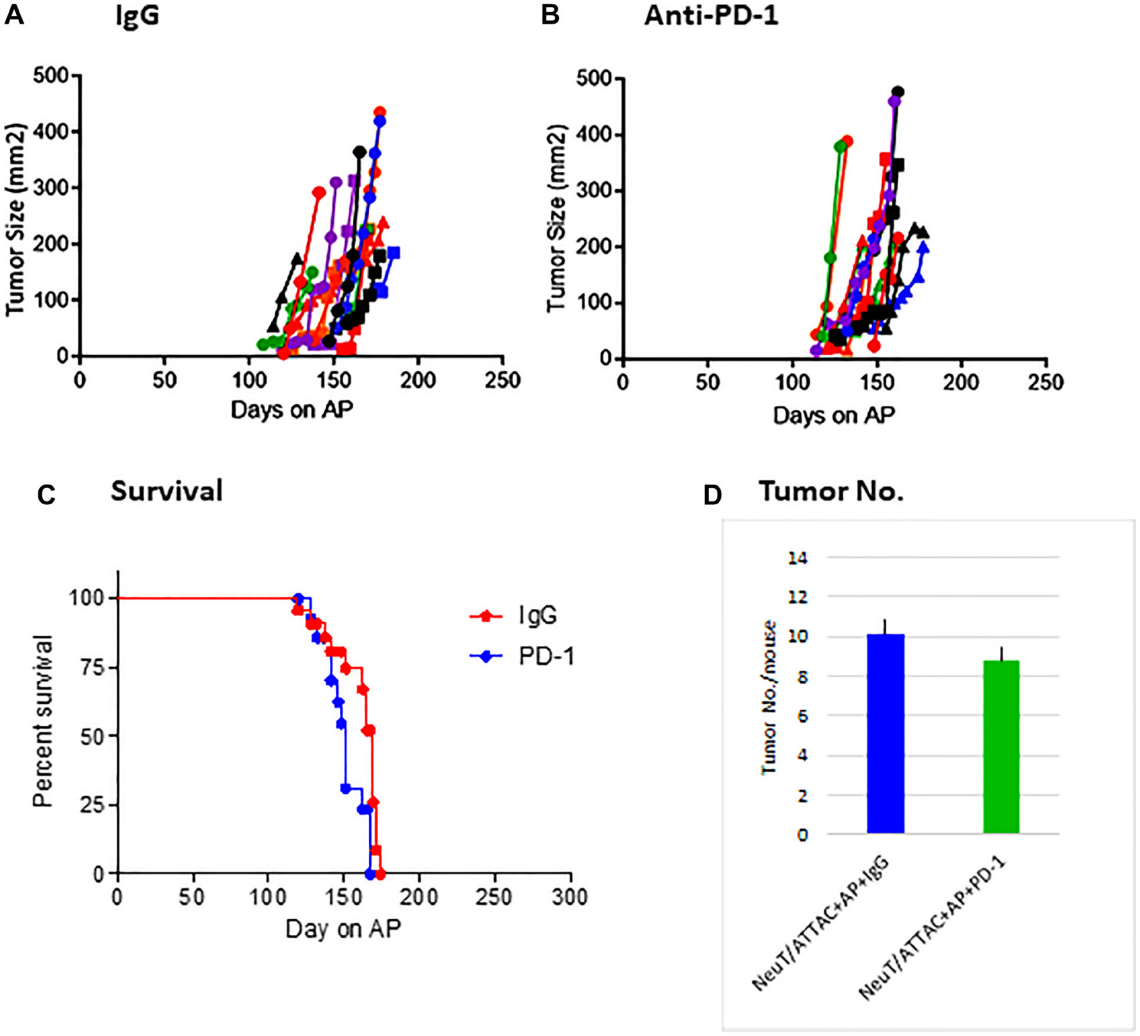
Effect of anti-PD-1 mAb treatment on tumorigenesis in NeuT/ATTAC mice. Tumor development in IgG-treated (**A**) and anti-PD-1-treated (**B**) mice over four months. Mice were treated triweekly by i.p. injection of 0.4 mg/kg AP21087 to induced fibrosis [21]. (**C**) Survival in NeuT/ATTAC mice. There was no significant difference between the IgG- and anti-PD-1-treated groups by the Mantel-Cox log-rank test. (**D**) Tumor multiplicity in NeuT/ATTAC mice after treatment with IgG or anti-PD-1. There was no significant difference (*P* > 0.05) between the IgG- and anti-PD-1-treated groups by the two-sided Student’s *t* test.

NeuT/ATTAC mice were used as a rigorous tumor model to test the efficacy of a murine-specific anti-PD-1 monoclonal antibody (mAb). Mice were treated with 100 μg of the mAb or isotype-specific IgG twice weekly for four months and tumor development, tumor multiplicity and survival determined (Figure 1). Anti-PD-1 treatment did not affect these parameters, and therefore a detailed analysis of changes in the tumors and the immune environment were assessed to determine possible mechanisms for this lack of responsiveness.

**Figure 2:**
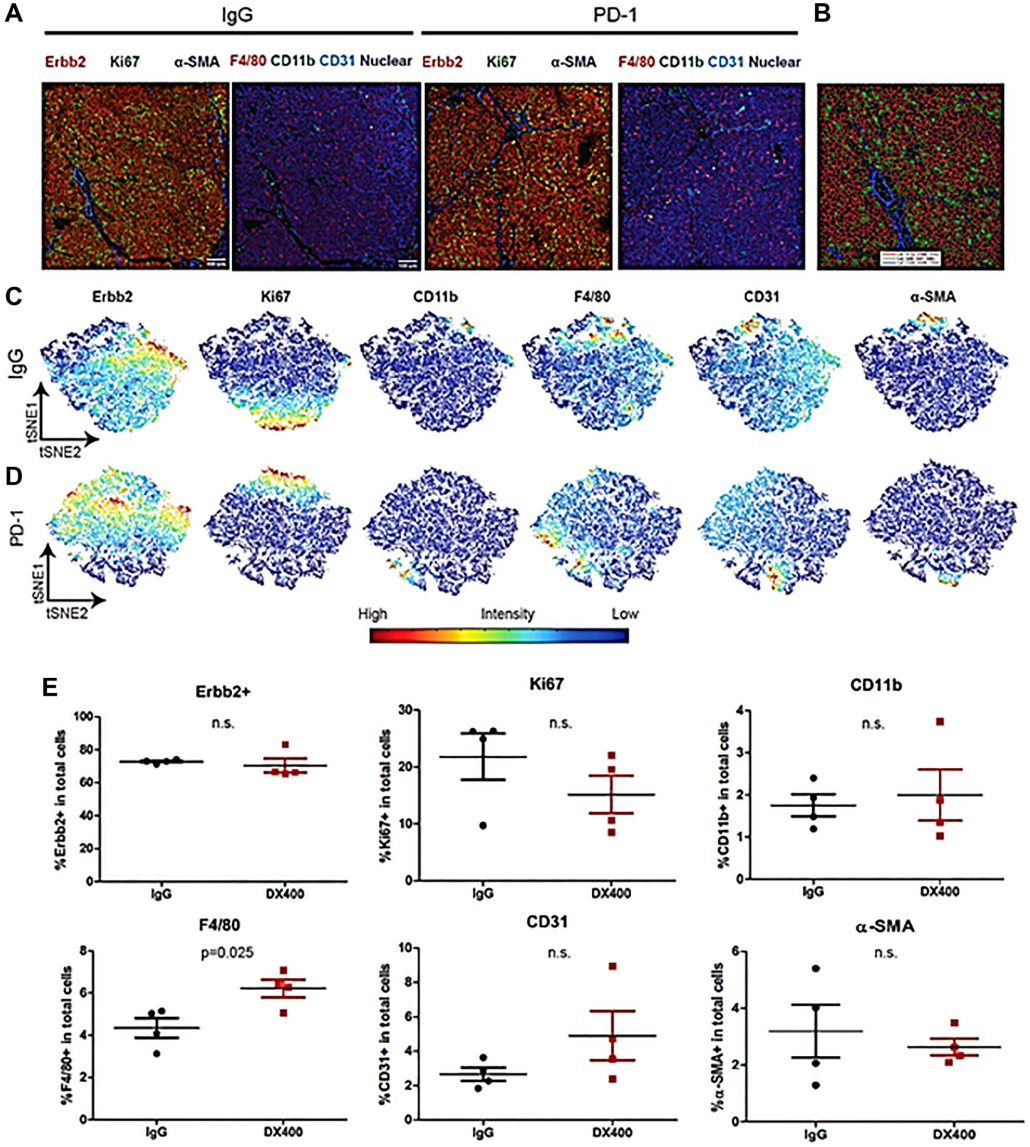
Imaging mass cytometry of tumors from NeuT/ATTAC mice after treatment with anti-PD-1 or IgG. Multiplex IHC and IMC was used to assess the effect of anti-PD-1 mAb treatment on the cellular composition of tumors from NeuT/ATTAC mice in which fibrosis was induced by AP21087 [21]. (**A**) Images of IgG and anti-PD-1-treated groups were obtained by staining with anti-Erbb2, −Ki67, −αSMA, −F4/80, −CD11b, and −CD31 antibodies. (**B**) Segmentation masks were generated for each image to visualize and quantify the cell types of interest into two dimensions [43]. (**C**, **D**) Erbb2^+^, Ki67^+^, CD11b^+^, F4/80^+^, CD31^+^ and αSMA^+^ cells were visualized in the IgG group and anti-PD-1 groups (*N* = 4 per group). (**E**) Quantification of the percentage of Erbb2^+^, Ki67^+^, CD11b^+^, F4/80^+^, CD31^+^ and αSMA^+^ cells from the IgG and anti-PD-1 groups. There was a significant increase in tumor-associated F4/80^+^ macrophages (*p* < 0.025).

IMC of tumors was used to evaluate changes in the TME (Figure 2). Similar cell types from IgG- and anti-PD-1-treated animals were grouped together (Figure 2A–2D). Ki67^+^ proliferating cells were visualized predominantly in Erbb2^+^ tumors, and anti-PD-1 mAb treatment did not alter the percentages of Erbb2^+^, Ki67^+^, CD11b^+^ monocytic myeloid cells, CD31^+^ endothelial cells or αSMA^+^ fibroblasts/pericytes; however, a significant increase in tumor-associated F4/80^+^ macrophages (*p* < 0.025) was observed (Figure 2E).

**Figure 3:**
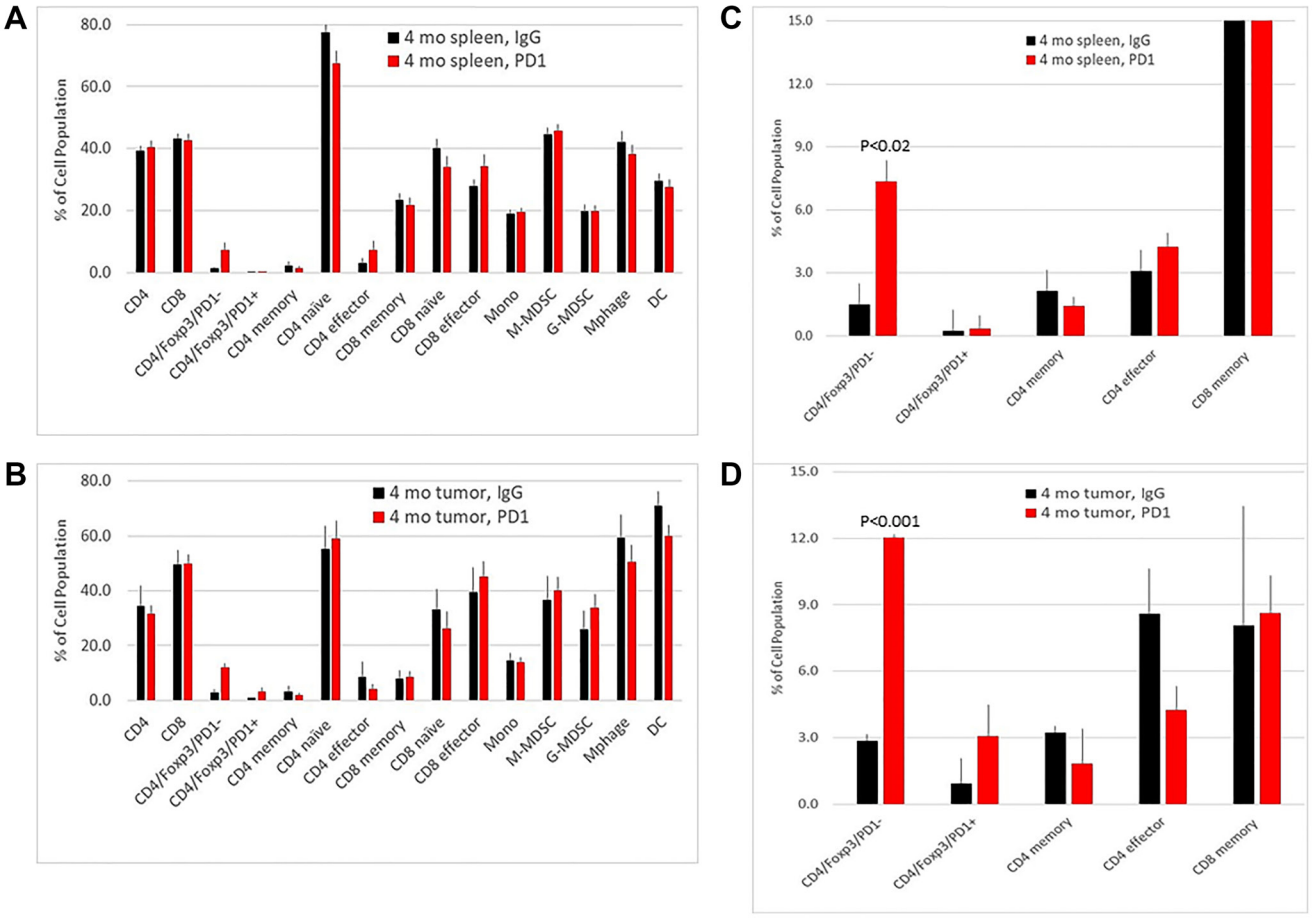
Flow cytometry of immune cell subsets in spleen and tumors from NeuT/ATTAC mice after treatment with IgG or anti-PD-1. FACS analysis of immune cells from spleen (**A**, **B**) and tumor infiltrates (**C**, **D**) after IgG and anti-PD-1 treatment. Panels B and D are presented on an expanded scale. There were significant increases in CD4^+^/Foxp3/PD-1^−^ Treg cells in spleen (*P* < 0.02) and tumors (*P* < 0.001). Statistical significance was determined by the two-tailed Student’s *t* test.

**Figure 4:**
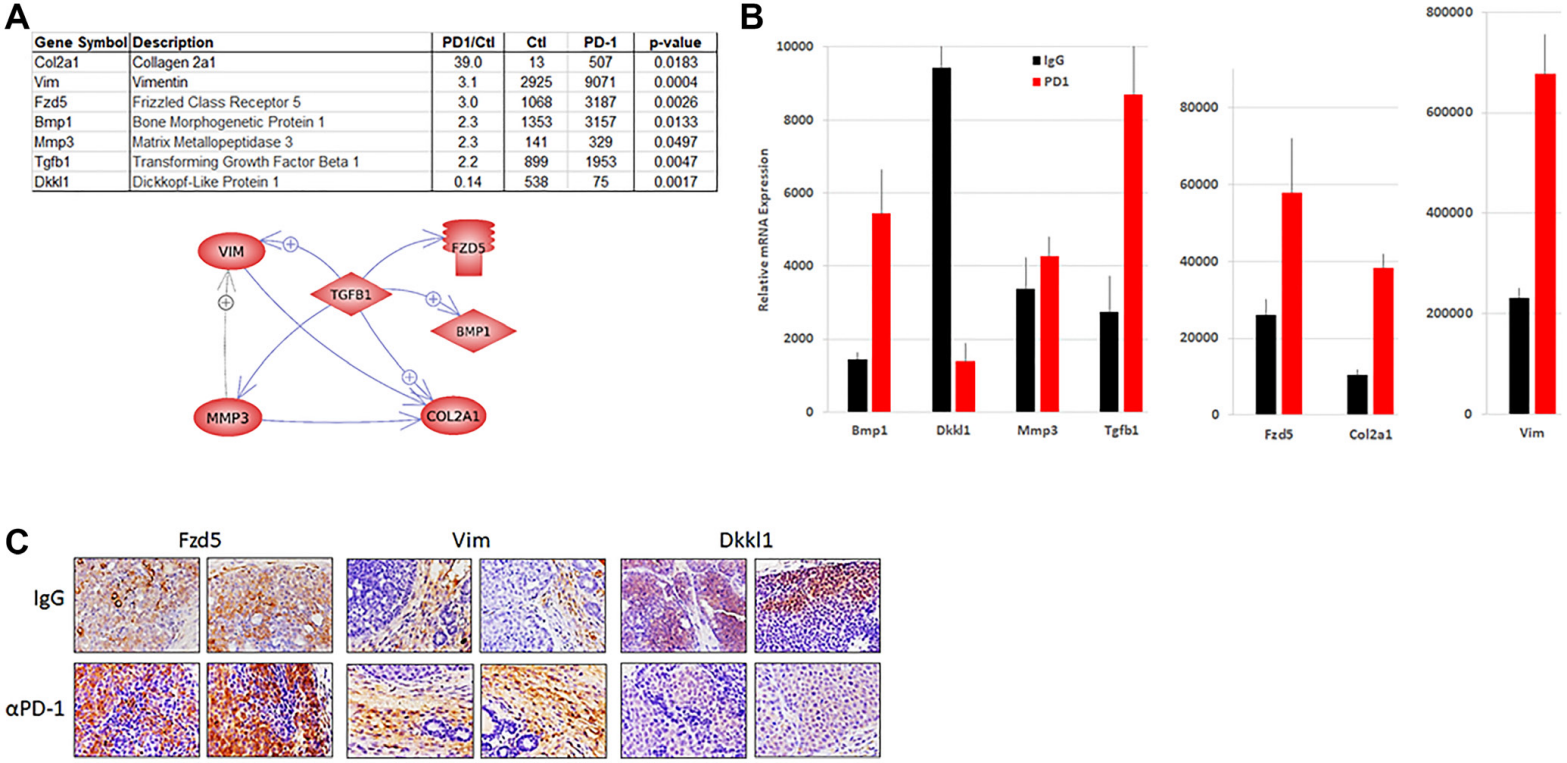
Wnt pathway expression in tumors from NeuT/ATTAC mice. RNA was prepared from mammary tumors from each group (*N* = 5) and pooled for RNAseq. (**A**) Wnt pathway genes that were upregulated in anti-PD-1-treated mice. Gene interactions were determined with Pathway Studio version 9.1. (**B**) qRT-PCR analysis of the genes in (A). (**C**) IHC of Wnt pathway components in tumors of NeuT/ATTAC mice.

Analysis of tumor infiltrating immune cells by FACS indicated a significant increase of Foxp3^+/^PD-1^−^ Treg cells, but no changes in the other cell subsets (Figure 3). To gain greater clarity about the processes associated with the insensitivity to anti-PD-1 therapy, transcriptomic analysis of the tumors was performed by RNAseq (Supplementary Table 1). Several processes were significantly upregulated, including myofibroblast differentiation, epithelial-to-mesenchymal transition (EMT), endothelial cell proliferation, ECM assembly, tumor growth and the Wnt pathway (Table 1). The latter pathway included increased expression of Fzd5, Lef1, Tgfb1, Bmp1, Mmp3, Col2a1 and Vim, and reduced expression of the Fzd5 inhibitor Dkkl1 (Figure 4A, 4B). Increased protein expression of Fzd5 and Vim and reduced expression of Dkkl1 were confirmed by IHC (Figure 4C).

**Table 1:** Transcriptomic analysis of the biological processes upregulated in tumors from NeuT/ATTAC+AP mice following anti-PD-1 treatment

Gene set seed	Total # of Neighbors	Overlap	Overlapping entities	*p*-value
Myofibroblast differentiation	669	9	VCAN;CCN2;MMP3;BGN;PLAUR;VIM;TERT;WNT5A;TGFB1	5.00E-10
Mesenchymal stem cell differentiation	850	9	VCAN;CCN2;COL2A1;PLAUR;CCND1;LEF1;TERT;WNT5A;TGFB1	5.15E-09
Canonical Wnt signaling pathway	2592	13	CCN2;MMP3;PLAUR;VIM;CCND1;LEF1;TERT;WNT5A;VCAN;COL2A1;BGN;FZD5;TGFB1	7.06E-09
Fibroblast proliferation	1278	10	VCAN;CCN2;MMP3;BGN;PLAUR;VIM;CCND1;TERT;WNT5A;TGFB1	1.43E-08
Endothelial cell proliferation	1766	11	CCN2;MMP3;PLAUR;VIM;CCND1;TERT;WNT5A;VCAN;BGN;BMP1;TGFB1	2.59E-08
Smooth muscle cell migration	749	9	VCAN;CCN2;MMP3;BGN;PLAUR;VIM;CCND1;WNT5A;TGFB1	3.75E-08
Fibrogenesis	1091	9	VCAN;CCN2;MMP3;BGN;PLAUR;VIM;CCND1;WNT5A;TGFB1	5.70E-08
ECM assembly	881	8	VCAN;CCN2;COL2A1;BGN;VIM;WNT5A;BMP1;TGFB1	1.52E-07
Tumor cell growth	2081	11	CCN2;MMP3;PLAUR;VIM;CCND1;LEF1;TERT;WNT5A;VCAN;BGN;TGFB1	1.69E-07
Macrophage infiltration	1086	9	CCN2;COL2A1;MMP3;BGN;PLAUR;VIM;TERT;WNT5A;TGFB1	9.12E-07

Shown are processes based on a ≥ 1.5× increase in RNA expression with a *p*-value < 0.05 (Supplementary Table 1) using Pathway Studio v. 9.

## DISCUSSION

One of the impediments to successful cancer therapy is the heterogeneity and cellular plasticity of the tumor and TME [22, 23]. Using the NeuT/ATTAC fibrotic breast cancer model, we show that its unresponsiveness to anti-PD-1 therapy is associated in part with a gene network connected to increased expression of components of the Wnt pathway (Figure 4). These results are consistent with a previous study implicating the Wnt pathway in the lack of T cell expansion and resistance to anti-PD-1 therapy [24]. Similarly, the Wnt pathway has been shown to drive Tgfb1 and Bmp1 signaling [25, 26] and the promotion of a collagen-rich fibrotic phenotype that excludes T cell infiltration of the tumor [27, 28]. Interestingly, although the insensitivity to anti-PD-1 treatment in NeuT/ATTAC mice was associated with an increase in TAM (Figure 2), tumors exhibited a reduction in Arg1 expression (Supplementary Table 1), which usually defines tissue macrophages with M1 polarization and sensitivity to immune checkpoint inhibitor therapy [29–31]. However, this may not reflect Arg1 expression in TAM, which do not exhibit the distinct functionality of tissue macrophages [32].

Although the present study was conducted in AP-21087-treated NeuT/ATTAC FVB mice and not in untreated mice, previous studies have shown that MMTV-NeuT Balb/c mice were insensitive to anti-PD-1 treatment [33] and failed to generate a cytotoxic T cell response to anti-erbB2 therapy [34], suggesting that the NeuT genotype is immune suppressed.

Lastly, among all of the tumor-infiltrating immune cell subsets, only Foxp3^+^/PD-1^−^ Treg cells were elevated (Figure 3), a subset known to be more immunosuppressive than Foxp3^+^/PD-1^+^ Treg cells [35] and a phenotype expected to be intrinsically insensitive to anti-PD-1 mAb.

Overall, the immune tolerant TME in NeuT/ATTAC mice was associated with tumor-infiltrating macrophages, Foxp3^+^/PD-1^-^ Treg cells as well as upregulation of the Wnt signaling pathway, which may provide further insights into the therapeutic options that may enhance immune checkpoint therapy.

## MATERIALS AND METHODS

### Animals

MMTV-NeuT mice [36] were obtained from Jackson Labs (FVB-Tg(MMTV-Erbb2)NK1Mul/J) [37] and FAT-ATTAC mice on a C57BL/6 background were kindly provided by Dr. Philipp Scherer, University of Texas Southwestern [38, 39]. FAT-ATTAC mice were crossed into the FVB strain and subsequently with MMTV-NeuT mice to produce NeuT/ATTAC mice as previously described [21]. In brief, NeuT/ATTAC mice at 6 weeks of age were injected i.p. triweekly with 0.4 mg/kg AP20187 throughout tumor development to induce fibrosis [21]. At 8 weeks of age, mice were injected i.p. twice weekly with 100 μg of the anti-PD-1 monoclonal antibody (mAb) or a matching isotype-specific IgG mAb for four months. This dose achieved a plasma level of 36 ± 3.6 μg/ml (mean ± S.E., *N* = 21), suggesting even distribution in total blood volume (unpublished results). All treatments and tumor measurements were carried out by Carlos Benitez and Maria Idalia Cruz under the auspices of the Animal Shared Resource.

### Fluorescence-activated cell sorting (FACS)

Tumor and spleen were removed and digested with collagenase D (Roche) and the cell suspension filtered, washed and erythrocytes lysed before analysis of 1 × 10^6^ cells by FACS as previously described [15]. Viable cells were determined with the Live/Dead Fixable Dead Cell Stain Kit (Invitrogen) and excluded from analysis, and non-specific binding was blocked with Fc antibody CD16/32 (Biolegend). Cells were sorted for CD45^+^ cells and subsequently for macrophages (F4/80^+^/MHCII^+^), G-MDSC (CD11b^+^/Gr-1^+^), M-MDSC (CD11b^+^/Ly6C^+^), dendritic cells (CD11c^+^/MHCII^+^), T cells (CD4^+^/CD8^+^), NK cells (CD45^+^/NK1.1^+^) and Treg cells (Foxp3^+^/PD-1) as previously described [15, 21]. Analysis was conducted by the Flow Cytometry and Cell Sorting Shared Resource using a BD LSRFortessa analyzer (BD Biosciences) and FCS Express 4 software (De Novo Software). Antibodies and their dilutions are listed in Supplementary Table 2.

### Histopathology and immunohistochemistry

Mammary tissue was excised and FFPE sections were prepared for antigen retrieval as previously described [40, 41]. Biotin-conjugated secondary antibodies were diluted in TBS containing 0.1% Tween-20 and incubated for 30 min at room temperature using ABC Vectastain (Vector Laboratories) and diaminobenzidine (Pierce). Slides were counterstained with Harris-modified hematoxylin (Thermo-Fisher, Inc.), dehydrated and mounted in Permount (Thermo-Fisher, Inc.). Antibodies and their dilutions are listed in Supplementary Table 2.

### RNAseq analysis

RNA was extracted, its quality assessed and RNAseq performed by Novagene as previously described [21, 42]. Raw data quality was checked using FastQC (v0.11.9), and adapter trimming of raw data was performed using Cutadapt (v3.5). The reference genome was downloaded from Ensembl mm10 release 99, and the reference genome index was built using Bowtie2 (v2.4.1) software. Paired-end trimmed reads alignment and raw read count calculation were performed using RSEM software (v1.3.1). Statistical analysis was performed using the DESeq2 package (v1.36.0) in R (v4.1). Genes with *p*-value < 0.05 were considered as differentially expressed and used as input for Gene Set Enrichment Analysis (GSEA) (v4.2.3, Broad Institute). RNAseq data were deposited in the GEO database under accession no. GSE215964.

### Quantitative real-time polymerase chain reaction (qRT-PCR)

Total RNA (1 μg) from each of 3 samples per group was reverse transcribed using the Omniscript RT kit (Qiagen) as previously described [21, 41]. PCR was performed in triplicate using an ABI-Prism 7700 (Applied Biosystems) and SYBRGreen I detection (Qiagen) according to the manufacturer’s protocol. Amplification using the appropriate primers was confirmed by ethidium bromide staining of the PCR products on an agarose gel. The expression of each target gene was normalized to GAPDH and is presented as the ratio of the target gene to GADPH expression calculated using the formula, 2^−ΔCt^, where ΔCt = Ct^Target^−Ct^18s^ [41]. RT-PCR primers are listed in Supplementary Table 3.

### Imaging mass cytometry (IMC)

Multiplex IHC and IMC was used to assess the effect of anti-PD-1 mAb treatment on the cellular composition of tumors from NeuT/ATTAC mice (*N* = 4 per group) after induction of fibrosis by AP21087 [21]. Twenty-four tumor, stromal and immune cell markers were tested, and six positive signals were obtained (Figure 2A). Segmentation masks were generated for each image to enable single cell data extraction with histoCAT software to visualize and quantify the cell types of interest [43]. T-SNE, a multidimensional reduction tool, was used to generate single cell data into two dimensions. A list of antibodies is provided in Supplementary Table 2.

### Statistical analysis

Statistical significance of means ± S.E. were evaluated using the two-tailed Student’s *t* test at a significance of *P* < 0.05. Survival data were analyzed using the Mantel-Cox log-rank test and tumor growth by the unpaired two-tailed Student’s *t* test at a significance of *P* < 0.05 using Prism GraphPad software.

## SUPPLEMENTARY MATERIALS




