# Supplementary material for: Resistance of MMTV-NeuT/ATTAC mice to anti-PD-1 immune checkpoint therapy is associated with macrophage infiltration and Wnt pathway expression
Source: Oncotarget. 2022 Dec 20;13:1350–8. doi: 10.18632/oncotarget.28330 (PMC9765860; doi:10.18632/oncotarget.28330)
Supplement: Supplementary file 1 [file oncotarget-13-28330-s001.pdf]

Resistance of MMTV-NeuT/ATTAC mice to anti-PD-1 immune checkpoint therapy is associated with macrophage infiltration and Wnt pathway expression

SUPPLEMENTARY MATERIALS

Supplementary Table 1: RNAseq results of genes with ≥1.5-fold change, *p*-value ≤ 0.05 and a raw score ≥300. See Supplementary Table 1

Supplementary Table 2: Antibodies for IHC, IMC and FACS

**IHC**				
**Antibody**	**Catalog#**	**Source**	**Lot#**	**Dilution**
TGF-beta1	MA1-21595	Invitrogen		
Plaur (Urokinase)	MA5-32586	Invitrogen	XF3611962C	1/250
CyclinD1	701421	Invitrogen	XF3614729	1/400
Vimentin	ab8978	Abcam	QI224056	1/1000
Dkkl1	102-12780	RayBiotech		1/500
Frizzled5	ab75234	Abcam	GR309195-44	1/400
Lgr6	ab214325	Abcam	GR3237703-16	1/125
**FACS**				
**Myeloid**	**Catalog#**	**Source**	**Conjugate**	**Clone**
Ly6g	127628	Biolegend	BV421	1A8
CD11b	563168	BD	BV711	M1/70
F4/80	123141	Biolegend	BV785	BM8
Ly6C	553104	BD	FITC	AL-21
CD8a	100732	Biolegend	PerCPCy5.5	53-6.7
CD86	105008	Biolegend	PE	GL-1
I-A/I-E	25-5321-82	eBioscience	PE-Cy7	M5/114.15.2
CD80	102716	Biolegend	A647	16-10A
CD11c	565872	BD	APC-R700	N418
CD45	103116	Biolegend	APC-Cy7	30-F11
**Lymphocytes**				
CD49b	563063	BD	BV421	DX5
CD335	562850	BD	BV421	29A14
CD25	740714	BD	BV711	PC61
CD44	563736	BD	BV785	IM7
CD4	553729	BD	FITC	GK1.5
CD3e	551163	BD	PerCPCy5.5	145-2C11
TCRb	560657	BD	PerCPCy5.5	H57-597

CD62L	553151	BD	PE	MEL-14
PD-1	109110	Biolegend	PE-Cy7	RMP1-30
Foxp3	17-5773-82	eBioscience	APC	FJK-16s
CD8	564983	BD	APC-R700	53–6.7
CD45	103116	Biolegend	APC-Cy7	30-F11
Live/Deadstain				

IMC

Antibody	Metal	Catalog#	Source	Clone	Conc. (ug/ml)
CD11b	149Sm	3149028B	Fluidigm	EPR1344 (cross)	2.5 (1:200)
CD31	150Nd	77699S	R&D	D8V9E	2.5 (1:200)
F4/80	158Gd	70076S	R&D	D2S9R	5 (1:100)
Erbb	161Dy	ab16901	Abcam	3B5	0.2
Ki67	168Er	3168022D	Fluidigm	B56 (cross)	5 (1:100)
a-SMA	176Yb	14-9760-82	Abcam	1A4	2.5 (1:200)

Supplementary Table 3: List of primers for qRT-PCR analysis

Gene accesion#	Primer name	Sequence	Amplicon size (bp)
Bmp1	FWD	GAC AAC TCGGTA CAGAGGAAA G	109
NM_001360021	REV	CGA ACTGGGCATGGGAATAA	
Col2a1	FWD	GCA GAATGGGCAGAGGTATAA	116
NR_177066	REV	AGTCTGGGTCTTCACAGATAATG	
Dkkl1	FWD	ATGTCGACTCTC AGC AGAAC	99
NM_015789	REV	GGTCTCGTAGCAGGTCATTT	
Fzd5	FWD	CTGTGTGTGTCACTGGGATT	99
NM_022721	REV	GCC GGTAGTCTC ATAGTGAATG	
Gapdh	FWD	GGC AAATTC AACGGC ACA	93
NM_001289726.1	REV	GTTAGTGGGGTCTCGCTCCTG	
Mmp3	FWD	GGA CCAGGGATTAATGGAGATG	92
NM_010809	REV	TGA GCA GCA ACC AGGAATAG	
Tgfb1	FWD	CTGAACCAA GGAGAC GGAATA C	101
NM_011577	REV	GGGCTGATCCCGTTGATTT	
Vim	FWD	CCC TGA ACC TGAGAGAAA CTAAC	93
NM_011701	REV	CTC TGGTCTCAA CCGTCTTAATC	
